# The relation between Parkinson’s disease and non-steroidal anti-inflammatories; a systematic review and meta-analysis

**DOI:** 10.3389/fphar.2024.1434512

**Published:** 2024-07-31

**Authors:** Amal Mohammad Badawoud, Lashin Saad Ali, Mahmoud S. Abdallah, Ramy M. El Sabaa, Mostafa M. Bahaa, Thanaa A. Elmasry, Eman Wahsh, Mohamed Yasser, Nashwa Eltantawy, Mamdouh Eldesoqui, Manal A. Hamouda

**Affiliations:** ^1^ Department of Pharmacy Practice, College of Pharmacy, Princess Nourah Bint Abdulrahman University, Riyadh, Saudi Arabia; ^2^ Department of Basic Medical Science, Faculty of Dentistry, Al-Ahliyya Amman University, Amman, Jordan; ^3^ Physiology Department, Faculty of Medicine, Mansoura University, Mansoura, Egypt; ^4^ Department of Clinical Pharmacy, Faculty of Pharmacy, University of Sadat City (USC), Sadat City, Egypt; ^5^ Department of PharmD, Faculty of Pharmacy, Jadara University, Irbid, Jordan; ^6^ Clinical Pharmacy Department, Faculty of Pharmacy, Menuofia University, Shibin Al Kawm, Egypt; ^7^ Pharmacy Practice Department, Faculty of Pharmacy, Horus University, New Damietta, Egypt; ^**8**^ Pharmacology and Toxicology Department, Faculty of Pharmacy, Tanta University, Tanta, Egypt; ^9^ Pharmacology and Toxicology Department, Faculty of Pharmacy, Sinai University, Arish, Egypt; ^10^ Department of Pharmaceutics, Faculty of Pharmacy, Port Said University, Port Said, Egypt; ^11^ Department of Pharmaceutics and Industrial Pharmacy, Faculty of Pharmacy, Horus University, New Damietta, Egypt; ^12^ Pharmacy Practice Department, Faculty of Pharmacy, Heliopolis University, Cairo, Egypt; ^13^ Department of Basic Medical Sciences, College of Medicine, AlMaarefa University, Riyadh, Saudi Arabia; ^14^ Department of Anatomy and Embryology, Faculty of Medicine, Mansoura University, Mansoura, Egypt

**Keywords:** NSAIDs (non-steroidal anti-inflammatory drugs), Parkinson’s disease, aspirin, ibuprofen, neuroprotecion

## Abstract

**Background:** Parkinson’s disease (PD) is a neurological condition that typically shows up with aging. It is characterized by generalized slowness of movement, resting tremor or stiffness, and bradykinesia. PD patients’ brains mostly exhibit an increase in inflammatory mediators and microglial response. Nevertheless, a variety of non-steroidal anti-inflammatory medications (NSAIDS) offered neuroprotection in animal models and preclinical trials.

**Aim:** The current systematic review and meta-analysis were designed to try to resolve the debate over the association of NSAID use with the development of PD because the results of several studies were somehow contradictory.

**Methods:** An intense search was performed on Scopus, PubMed, and Web of Science databases for articles relating the incidence of PD to the use of NSAIDs. Statistical analysis of the included studies was carried out using Review Manager version 5.4.1 by random effect model. The outcome was identified as the development of PD in patients who were on NSAIDs, ibuprofen only, aspirin only, and non-aspirin NSAIDs. This was analyzed using pooled analysis of odds ratio (OR) at a significance level of ≤0.05 and a confidence level of 95%. A statistically significant decreased risk of PD was observed in patients taking NSAIDs, Ibuprofen, and non-aspirin NSAIDs.

**Results:** The ORs of PD occurrence in patients who took NSAIDs, Ibuprofen, and non-aspirin NSAIDs were 0.88 [95% CI (0.8–0.97), *p* = 0.01], 0.73 [95% CI (0.53–1), *p* = 0.05] and 0.85 [95% CI (0.75–0.97), *p* = 0.01]. Meanwhile, the risk of PD in patients who took aspirin was not statistically significant.

**Conclusion:** In conclusion, Ibuprofen, non-aspirin NSAIDs, and other types of NSAIDs could be associated with a reduction in PD risk. However, there was no association between aspirin intake and the development of PD.

## 1 Introduction

Parkinson’s disease (PD) is a neurological condition that typically shows up with aging or longevity. In addition to generalized slowness of movement (bradykinesia), there are at least two other symptoms of resting tremor or stiffness. Other signs include loss of smell, difficulty sleeping, mood swings, excessive salivation, constipation, and irregular, frequent limb movements during sleep (REM behavior disorder). The prevalence of PD among those aged 60 years and older is 1%. The disease is associated with the presence of Lewy bodies and the degeneration of dopaminergic neurons in the substantia nigra. Most of these diseases are idiopathic. Only 10% of cases especially in children have a genetic component (Alexoudi et al., 2018; Kabra et al., 2018; Mirpour et al., 2018; Zafar and Yaddanapudi, 2022).

Inflammation may play a role in the development of PD, as was earlier mentioned, according to a considerable body of evidence from both human samples and animal models. However, the precise cause of this reaction is still unknown. It’s possible that continuing neuronal cell death in PD leads to inflammation, but it’s also possible that misfolded—synuclein (Syn) has a direct impact. Peripheral inflammation and genes linked to PD risk suggest a crucial role in the chronic inflammatory response at the onset of this neurodegenerative condition, in addition to the well-known microgliosis and astrogliosis in PD brains (Pajares et al., 2020).

Nonsteroidal anti-inflammatory medicines (NSAIDs) are commonly used to alleviate pain, reduce swelling, relieve stiffness, and treat inflammation in the limbs (Driver et al., 2011). These drugs have extensive clinical usage. Besides their anti-inflammatory properties, NSAIDs have also garnered interest for their potential in preventing and treating Parkinson’s disease (Bornebroek et al., 2007). The usage of NSAIDs is associated with Parkinson’s disease and can be explained from a biological perspective. Neuroinflammation has been found to be associated with the development of Parkinson’s disease, and NSAIDs have been shown to offer neuroprotection in animal models (Herna et al., 2006). In animal models, a variety of NSAIDS offer neuroprotection, while PD patients’ brains exhibit an increase in inflammatory mediators and microglial response. The relationship between NSAID use and the risk of PD has gotten very little attention in observational research, despite a plethora of epidemiological evidence supporting their protective benefits in the development of Alzheimer’s disease. A reduction in the risk of PD has been linked by the majority of these studies to the use of non-aspirin NSAIDs. However, studies on animal models have demonstrated that mice can be protected from MPTP-induced striatal dopamine depletion by taking acetylsalicylic acid (aspirin) (Herna et al., 2006; Ton et al., 2006; Bornebroek et al., 2007; Driver et al., 2011; Manthripragada et al., 2011).

Contradictory findings were observed regarding the association between NSAIDs and PD disease development. For example, Gagne and Power (2010) showed that there was an association between the use of non-aspirin NSAIDs and the reduction of PD risk. This study also showed that there was no association between aspirin use and PD development. On the other hand, a study was conducted by Samii et al. (2009) on 11 observational studies and showed no effect of NSAIDs on developing PD. Due to the conflicting literature and the gap of knowledge, this systematic review/meta-analysis was designed to try to resolve the debate over the association of NSAID use with the development of PD as the results of several studies were somehow contradictory.

## 2 Materials and methods

### 2.1 Search strategy

Scopus, PubMed, and Web of Science databases were employed to search for articles relating to the risk of PD to the use of NSAIDs from inception till February 2024. The search strategy was by title, abstract, and keywords as follows: (non-steroidal anti-inflammatory OR NSAIDS OR Aspirin OR Ibuprofen OR Naproxen OR Diclofenac AND Parkinson*)

### 2.2 Eligibility criteria and screening

All studies describing the risk between NSAIDS and PD were included. Randomized controlled trials, case-control, and cohort studies were included in this study. We identified the PICO of the study. We included a population of any age and any follow-up time taking any NSAID as an intervention. We didn't put a restriction on the duration of aspirin intake, types of NSAIDs, or language of the study. The comparison group was the group who didn’t take any NSAID, and the outcome was the incidence of PD. We excluded case reports, case series, narrative or systematic reviews, and meta-analyses. In addition, we excluded conference abstracts and unpublished manuscripts to prevent the bias of results. We also excluded studies describing the risk of neurodegenerative disease other than PD or describing the risk of PD in drugs other than NSAIDs because they didn’t fit into the outcome of interest. Screening was done by 2 authors independently and any difference between them was referred to a third author. Title and abstract screening were done first, and eligible articles were screened by full text thereafter.

### 2.3 Data extraction

Two authors carried out data extraction independently using Excel sheets and any difference was resolved by a third author. We extracted main baseline data as study design, sample size, age and gender of cases and controls, duration of NSAID use, duration of PD, type of NSAID, and effect sizes whether odds ratio or risk ratio.

### 2.4 Quality assessment

Quality assessment of the included studies was done independently by 2 authors as well with a third author to revise it. We carried out the process using the Newcastle-Ottawa Scale for case-control, and cohort studies. It is a tool made of 8 questions as presented in Tables 1, 2, each of them can take a star except one question of comparability that can take 2 stars, so the maximum score is 9. A score of 0–3 means low quality, 4–6 means moderate quality and 7–9 means high quality.

**TABLE 1:** Newcastle-Ottawa scale for quality assessment of case-control studies.

Study ID	Is the case definition adequate?	Representativeness of the cases	Selection of the controls	Definition of controls	Comparability of cases and controls	Ascertainment of exposure	Same method of ascertainment for cases and controls	Nonresponse rate
Wahner 2007 (Wahner et al., 2007)	★	★	★	—	★★	★	—	★
Starhof 2019 (Starhof et al., 2020)	★	★	★	—	★★	—	—	—
Powers 2008 (Powers et al., 2008)	★	★	★	★	★	—	—	—
Manthripragada 2011 (Manthripragada et al., 2011)	★	★	★	★	★★	—	—	★
Hernan 2006 (Herna et al., 2006)	★	★	★	★	★★	★	—	—
Etminan and Suissa 2006 (Etminan and Suissa, 2008)	★	★	★	—	★	★	—	—
Driver 2010 (Driver et al., 2011)	★	★	★	—	★★	—	—	—
Ton 2006 (Ton et al., 2006)	★	★	★	—	—	★	★	★
Sung 2016 (Sung et al., 2016)	★	★	★	★	★	★	★	★
Hancock 2007 (Hancock et al., 2007)	★	★	★	★	★★	—	—	★
Becker 2011 (Becker et al., 2011)	★	★	★	★	★	—	—	—

**TABLE 2:** New-Castle Ottawa scale for cohort studies.

Study ID	Representative-ness of the exposed cohort	Selection of the non-exposed cohort	Ascertainment of exposure	Demonstration that outcome of interest was not present at start of study	Comparability	Assessment of outcome	Was follow-up long enough for outcomes to occur	Adequ-acy of follow up
Etminan 2008 (Etminan et al., 2008)	★	—	★	—	★★	★	★	★
Chen 2003 (Chen et al., 2003)	★	★	★	★	—	★	★	★
Chen 2005 (Chen et al., 2005)	★	★	★	—	★	★	—	—

### 2.5 Statistical analysis

We carried out statistical analysis of the included studies using Review Manager version 5.4.1 by random effect model. The outcome was the PD onset in patients taking NSAIDs, ibuprofen only, aspirin only, and non-aspirin NSAIDs. This was analyzed using pooled analysis of odds ratio at a significance level of 0.05 and confidence level of 95%. We assessed the heterogeneity using the I^2^ value and Q statistics with the *p*-value assessing the significance of present heterogeneity at 0.05.

### 2.6 Publication bias assessment

The publication bias assessment was checked for pooled studies according to Egger et al. (1997) Funnel plots were constructed to present the relationship between effect size and standard error.

## 3 Results

### 3.1 Database searching and screening

**FIGURE 1:**
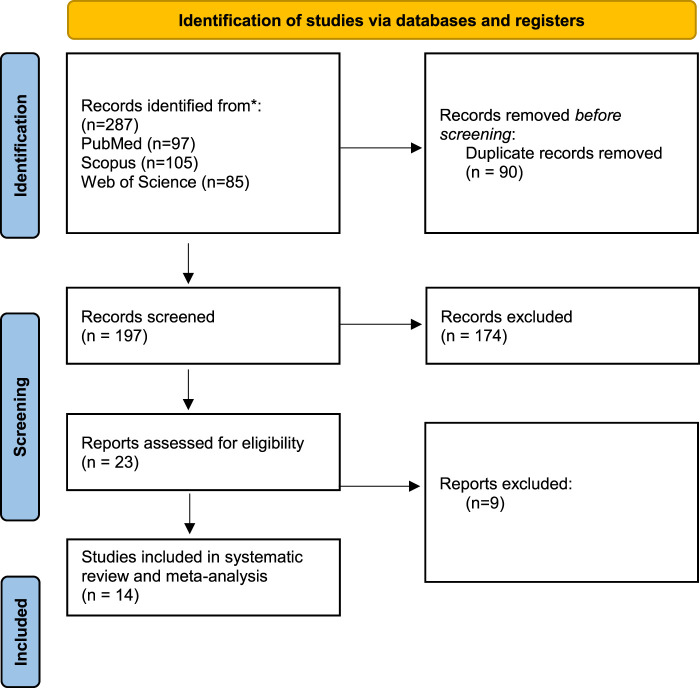
PRISMA flow diagram of database searching and screening.

Our search methodology resulted in 287 articles from the searched databases. After the removal of duplicates, 197 articles entered the screening process. By title and abstract screening, 23 articles were eligible for full-text screening which resulted in 14 articles entering our systematic review and meta-analysis (Figure 1).

### 3.2 Quality assessment

Of the 14 included studies, 11 were case-control, and 3 were cohort studies. Five case controls were of high quality and 6 were of moderate quality. Regarding cohort studies, 2 studies were of high quality and 1 was of moderate quality. This is shown in Tables 1, 2.

### 3.3 Baseline characteristics

Baseline data related to the included studies are summarized in the Table 3. We reported the adjustment done when calculating the odds ratio of PD occurrence and the lag time between NSAID and PD occurrence. We also reported the frequency of NSAID intake if mentioned.

**TABLE 3:** Baseline Characteristics of the included studies.

Study ID	Design	Sample Size, N		Age, mean (SD)		Gender (M/F)		Lag time, years	NSAID duration	Adjustment	Type of NSAID	Follow up
		Cases	Controls	Cases	Controls	Cases	Controls					
Wahner 2007 (Wahner et al., 2007)	Case-control	293	286	70.0 (10.7)	69.0 (12.9)	157/136	146/140	5	Regularly	Age at diagnosis, sex, race, geographical location, smoking, education	Aspirin, non-aspirin NSAIDs	—
Starhof 2019 (Starhof et al., 2020)	Case-control	155	7750	63.8 (9.3)	63.8 (9.3)	77/78	3850/3900	5	>2 prescriptions	Age, sex, place of residency, and COPD status	Non-aspirin NSAIDs	—
Powers 2008 (Powers et al., 2008)	Case-control	1,186	928	69.6 (30–97)	67.7 (25–94)	790/396	374/554	—	Ever	Age, race, sex and geographical location	NSAIDS	—
Manthripragada 2011 (Manthripragada et al., 2011)	Case control	1,931	9,651	72.2 (10.5)	72.2 (10.5)	1,121/810	5603/4048	5	Ever	Age, sex, comorbidities, COPD	Aspirin, non-aspirin NSAIDs	—
Hernan 2006 (Herna et al., 2006)	Case-control	1,258	6,638	70.7	68.7	765/493	3923/2715	—	Ever	Age, sex, treatment start date, clinic, smoking status, MI, arthritis	Aspirin, Non aspirin NSAIDs	—
Etminan and Suissa 2006 (Etminan and Suissa, 2008)	Case-control	1,259	12,590	73.9 (10.3)	73.9 (10.3)	692/567	5665/6925	1	Ever	Age, sex, antipsychotics, anti-RA medications	NSAIDs	—
Etminan 2008 (Etminan et al., 2008)	Cohort	5,010	692,068	—	—	—	—	—	Ever	age, sex, comorbidity (and use of antipsychotic medications	NSAIDs	6 years
Driver 2010 (Driver et al., 2011)	Case-control	565	2458	58.57 (40.1–85.0)	58.55 (40.0–85.0)	—	—	5	ever	Smoking, alcohol, BMI, exercise to sweat	Aspirin, Non aspirin NSAIDs	—
Chen 2003 (Chen et al., 2003)	Cohort	142,902		—	—	—	—	2	Ever	Age, smoking, caffeine and alcohol intake	Aspirin, Non aspirin NSAIDs	18 years
Ton 2006 (Ton et al., 2006)	Case-control	206	383	69.2 (9.0)	69.4 (8.6)	121/105	239/144	5	Ever	Age, sex, smoking, duration of enrolment, clinic	Aspirin, non aspirin NSAIDs, Ibuprofen	—
Sung 2016 (Sung et al., 2016)	Case-control	—	—	—	—	—	—	—	Ever	sex, age, and comorbidities, and NSAID use	NSAIDs	—
Chen 2005 (Chen et al., 2005)	Cohort	—	—	—	—	—	—	—	≥1/day	Age, sex, smoking, vitamin use, coffee, arthritis, other analgesics	Aspirin., ibuprofen, other NSAIDs	9 years
Hancock 2007 (Hancock et al., 2007)	Case-control	356	317	66.1 (10.7)	58.1(11.6)	235/121	139/178	—	Ever	Age at examination, sex	NSAIDs	—
Becker 2011 (Becker et al., 2011)	Case-control	4,026	15,969	60–80	60–80	2364/1662	9362/6607	—	Ever	BMI, smoking and comorbidities	Aspirin, Non aspirin NSAIDs, Ibuprofen, other NSAIDs	—

### 3.4 Statistical analysis

**FIGURE 2:**
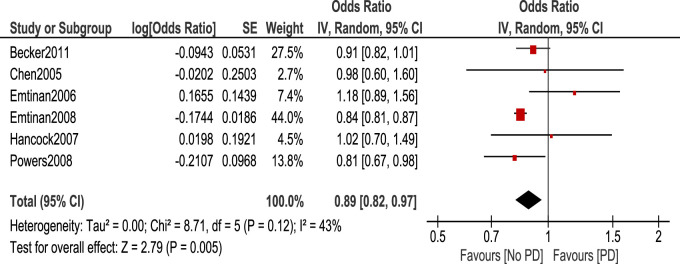
Forest plot of PD risk in patients who took NSAIDs.

Statistical tests were classified into 4 categories: NSAIDs, ibuprofen, Aspirin, and Non-aspirin NSAIDs. We calculated the odds ratio of PD occurrence in each case using the random effect model. Regarding NSAID use, the odds ratio of PD occurrence in patients who took NSAIDs was 0.89 (95% CI: 0.82–0.97, *p* = 0.005) (Figure 2). This shows a statistically significant decreased risk of PD in patients taking NSAIDs.

**FIGURE 3:**
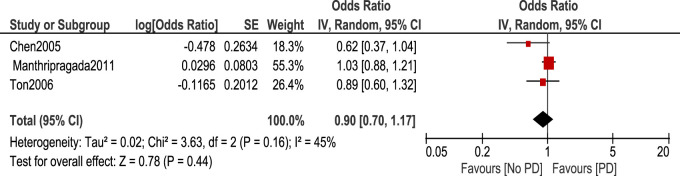
Forest plot of PD risk in patients who took Ibuprofen.

Regarding the use of ibuprofen, it is shown in Figure 3 that there was no statistically significant association between ibuprofen intake and risk of PD [OR 0.9, 95% CI (0.7–1.17), *p* = 0.44] with heterogeneity measured by I^2^ = 45%, *p* = 0.16.

**FIGURE 4:**
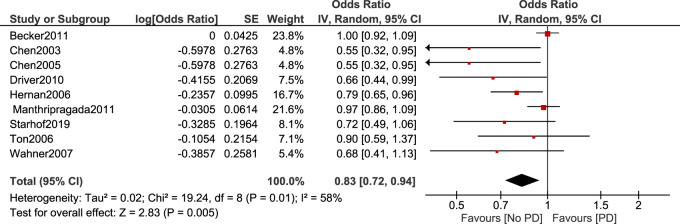
Forest plot of PD risk in patients who took Non-aspirin NSAIDs.

**FIGURE 5:**
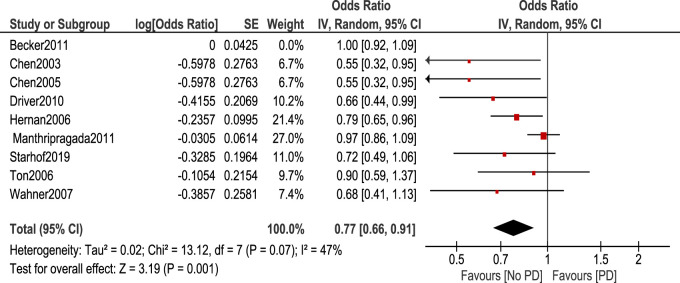
Leave one out, a meta-analysis of PD risk in Non-aspirin NSAIDs use.

Non-aspirin NSAIDs were associated with a lower risk of PD. [OR 0.83, 95% CI (0.72–0.94), *p* = 0.005] Heterogeneity was of I^2^ = 58%, *p* = 0.01 (Figure 4). Sensitivity analysis by leave one out method was done and heterogeneity became insignificant (I^2^ = 47%, *p* = 0.07) after removing Becker et al. (2011). Odds ratio became 0.77, 95% CI [0.66–0.91], *p* = 0.001 (Figure 5).

**FIGURE 6:**
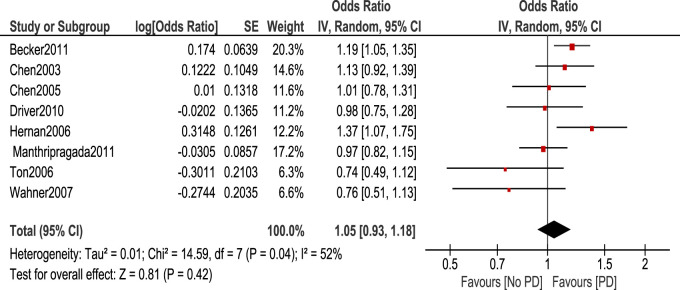
Forest plot of PD risk in patients who took Aspirin.

The association between aspirin and PD risk was null as shown in the Figure 6.

### 3.5 Risk of publication bias assessment using Egger’s test and funnel plots

**FIGURE 7:**
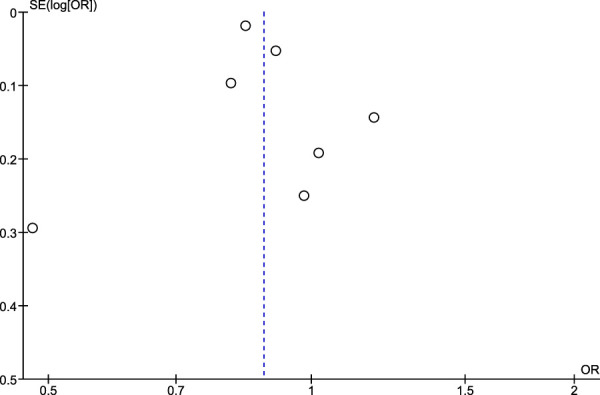
Funnel plot for risk of bias in NSAID users.

**FIGURE 8:**
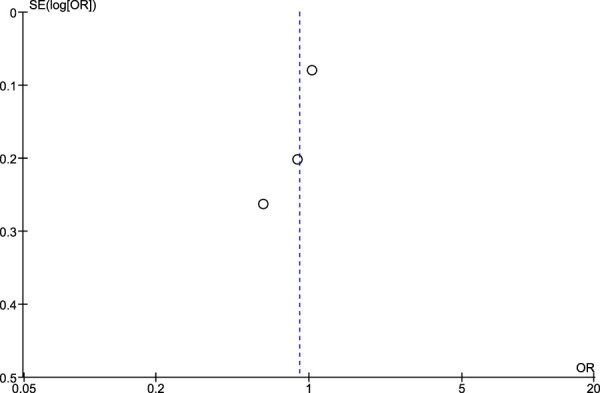
Funnel plot for risk of bias in ibuprofen users.

**FIGURE 9:**
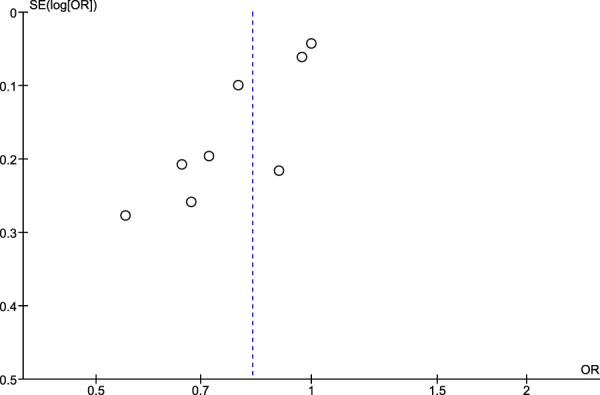
Funnel plot for risk of bias in non-aspirin-NSAIDs users.

**FIGURE 10:**
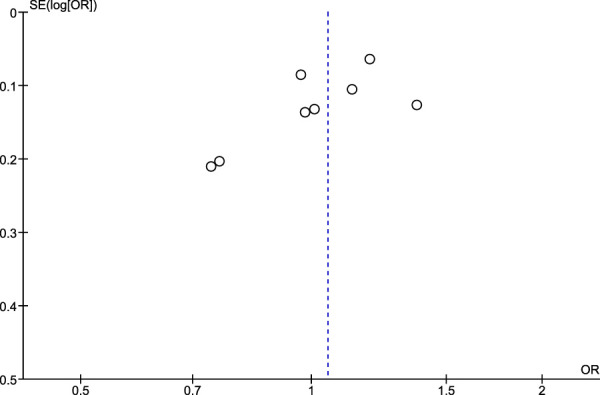
Funnel plot for risk of bias in aspirin users.

Regarding the risk of publication bias assessed by Egger’s test and funnel plots, it is seen to be low in NSAIDs and aspirin while it is moderate in non-aspirin NSAIDs and ibuprofen. A low risk of bias in NSAIDs and aspirin is associated with low heterogeneity in both, while a moderate risk of bias in non-aspirin NSAIDs is associated with moderate heterogeneity, however, the funnel plot isn't extremely accurate in the ibuprofen analysis due to a low number of included studies (Figures 7–10).

## 4 Discussion

Several studies including observational or systematic reviews and meta-analyses addressed the association between NSAIDs and the development of PD. Our study is considered among the most comprehensive since we targeted all available recent evidence and because of the categories we made. Our study included 17 studies which will be published until January 2024.

Our findings showed that there is a statistically significant reduction of PD risk in patients taking non-aspirin NSAIDs, and NSAIDS other than aspirin and ibuprofen. However, there was no statistically significant association between aspirin, and ibuprofen and the development of PD. Our findings are consistent with a meta-analysis conducted by Gagne and Power (2010) which showed the association between the use of non-aspirin NSAIDs and the reduction of PD risk. This study also showed that there is no association between aspirin use and PD development as shown by our findings. This study was conducted on 6 observational studies, so our study included more studies and in contrast to our analysis method by odds ratio, it was conducted using risk ratio. A similar inclusion of studies that observed the occurrence of PD some years after NSAID use was applied in this study as our inclusion criteria. This was done since it is improbable that exposures discovered within a year of PD diagnosis have an impact on disease incidence because PD initiation likely occurs long before the onset of symptoms (Rathore et al., 2009).

Another study done by Samii et al. (2009) on 11 observational studies showed that no effect of NSAIDs on developing PD which is inconsistent with our studies. It was also shown by 4 studies that risk reduction of PD had been observed in 4 studies in men compared to women, however, this is not enough to produce an evident protective role of NSAIDs from PD. This study also showed that ibuprofen is associated with a reduction of PD risk with a pooled risk ratio of 0.76 (95% CI 0.65, 0.89) through data from 3 studies. This study also showed that there was no statistically significant association between aspirin use and the development of PD through pooled analysis of six studies with a risk ratio of 1.08 (95% CI 0.93, 1.26) (Samii et al., 2009). This shows similar findings to our study as we found no statistically significant association between aspirin use and the development of PD. [OR 1.05. 95% CI (0.93–1.18)], *p* = 0.42.

Another systematic review by Alharbi et al. (2020) was conducted using 5 observational studies. It compared PD risk in aspirin users and non-users in addition to a comparison between PD risk in NSAID users and non-users. Findings regarding aspirin were different from our findings and those of other mentioned studies. A statistically significant rise in PD prevalence was observed among aspirin users compared to aspirin non-users with OR = 5.98 (95% CI = 1.743–20.547), *p* = 0.004. This is inconsistent with our study which showed no association between aspirin use and risk of PD. This study used the same effect estimate used by us (odds ratio), however the difference in results can be attributed to a greater number of studies involved in our systematic review and meta-analysis as we used a total of 10 studies to determine the relation between aspirin and PD while Alharbi et al. (2020) used only 5 studies so we provide more comprehensive and more up to date evidence. Alharbi et al. (2020) observed no statistically significant association between NSAID use and the development of PD using 5 studies as well with OR = 1.18 (95% CI = 0.580–2.436), *p* > 0.05 (Alharbi et al., 2020). This is inconsistent with our findings as we observed a statistically significant association between the use of NSAIDs and non-aspirin NSAIDs and the development of PD with an odds ratio of 0.89 (95 CI: 0.82–0.97, *p* = 0.005) and OR 0.83, 95% CI [0.72–0.94], *p* = 0.005 respectively.

The previous three meta-analyses had various limitations that should be addressed, and this supports the need for a further meta-analysis. Gagne and Power (2010) and Samii et al. (2009) were published in 2010 and 2009, respectively so they included a limited number of studies. Due to the recently published articles, a new meta-analysis should be done to include further findings.

Alharbi et al. (2020) showed an increased risk of PD in people taking aspirin however, many limitations exist in this meta-analysis such as incorrect OR estimates in addition to missing the inclusion of many studies investigating the risk of PD in people taking aspirin such as Chen et al. (2005), Driver et al. (2011), Wahner et al. (2007), Manthripragada et al. (2011), and Ton et al. (2006).

A study done by Poly et al. (2019) on 17 observational studies found that no statistically significant association exists between the use of NSAIDs and the development of PD with a risk ratio of 0.95 (95% CI 0.860–1.048), *p* = 0.304. This is inconsistent with our findings, and this can be attributed to various reasons. Firstly, in our analysis, we included studies that used adjusted effect estimates to lessen the risk of confounding bias associated with various factors in the primary studies such as age, gender, other comorbidities, disease duration, duration of NSAID use, etc. Secondly, in the part of NSAIDs analysis, we included studies that mentioned NSAIDs as a whole without the inclusion of studies investigating aspirin alone, ibuprofen alone, or non-aspirin NSAIDs alone. Thirdly, there is a major limitation in Poly et al. (2019)’s analysis as they included the hazard ratio and odds ratio in the same meta-analysis to produce risk ratio which is not appropriate statistically and could produce inaccurate results. Moreover, this study is entitled with elderly population, however, it included patients older than 18 years of age which is another major limitation. Furthermore, there exist other factors that may cause differences in results such as the difference in the used effect estimate as they used risk ratio not odds ratio. In addition to this, the risk ratio is less than 1 and the upper interval approaches 1 (Poly et al., 2019).

This study also showed that aspirin use is slightly associated with an increased risk of PD with RR 1.10, 95% CI 1.004–1.218, *p* = 0.04 which is inconsistent with our findings (no association) but consistent with the findings of Alharbi et al. (2020); Poly et al. (2019). Different from Poly et al. (2019), our study included a subgroup of non-aspirin NSAIDs which was observed to be associated with a decreased risk of PD exposure. Moreover, we included aspirin as a subgroup and the association was null. However, Poly et al. (2019) found an increased risk. Although the two studies included ten studies, we did n’t include Bower et al. (2006) as we excluded conference abstracts to include the high-quality peer-reviewed papers which increases the quality of the meta-analysis as well as that the full-text of the abstract wasn’t accessible. Moreover, there exist other factors that lead to differences in findings between us and Poly et al. (2019) as mentioned earlier.

Our results come in agreement with *in vitro* and *in vivo* studies. Several animal studies reported the efficacy of NSAIDs’ ability to reduce the risk of PD (Sánchez-pernaute et al., 2004; Wang et al., 2005; Parepally et al., 2006). The mechanism of NSAIDs regarding PD risk is not obvious till now. NSAIDs are frequently employed as the initial therapy option for inflammatory conditions and pain management. Recent research has demonstrated that the pharmacological benefits of NSAIDs are attributed to the inhibition of COX, and these medications have positive impacts in managing several neurological conditions (Sánchez-pernaute et al., 2004). Indomethacin removes nitric oxide free radicals, fenamate NSAIDs enhance GABA-A receptor function, and acetylsalicylic acid prevents the movement of NF-kB. Microglia, being a significant producer of prostaglandins (PGs), are thus seen as a suitable focus for neurological processes in the central nervous system (Terzi et al., 2018). The activation of PGE2 takes place during inflammation. Inflammatory activation by tumor necrosis factor (TNF)-α and interleukin (IL)-1β induces the production of COX-2 and membrane-associated PGES-1. Furthermore, cytosolic PGES is consistently linked with COX-1 (Terzi et al., 2018). COX-2 is the main factor responsible for the synthesis of PG in both acute and chronic inflammation. Selective COX-2 inhibitors are frequently employed to inhibit the production of PGE2 in microglial cells. Furthermore, COX-2 is stimulated in excitatory forebrain neurons within the brain. However, it is primarily increased in microglial cells both in laboratory settings (*in vitro*) and in living organisms (*in vivo*), and it is also influenced by the expression of mPGES-1 by microglia. Peroxisome proliferator-activated receptor-gamma (PPAR-γ) belongs to a set of nuclear receptors that can be activated by synthetic agonists, including NSAIDs such as indomethacin, ibuprofen, and diclofenac (Parepally et al., 2006). PPAR-γ synthetic agonists regulate brain inflammation and the viability of other neural cells. Additionally, they are involved in many pathways related to microglia, peripheral macrophages, and lymphocytes. Research indicates that NSAIDs have neuroprotective properties via inhibiting microglia in brain illnesses through a PPAR-γ-dependent mechanism (Terzi et al., 2018).

Our study provides comprehensive evidence with a large number of participants from different observational studies. Adjustment to different factors such as age, gender, smoking, and comorbidities was done so confounding is controlled with a large percentage. We defined NSAIDs into different categories including aspirin, ibuprofen, and non-aspirin NSAIDs.

Some limitations include the use of observational studies and the absence of experimental ones due to limited available data. Some studies reported a lag time of 5 years and some of less years between PD and NSAID use so this can result in bias. Moreover, PD can develop through a variety of mechanisms. But the focus of this study is solely on the inflammation-related mechanism.

We recommend future experimental studies defining the period between drug intake, its dose, and its type. Population should also be defined to explain the main risk.

## 5 Conclusion

Ibuprofen, non-aspirin NSAIDs, and other types of NSAIDs are associated with a reduction in PD risk, however, there was no association between aspirin intake and the development of PD.

## Data Availability

The raw data supporting the conclusions of this article will be made available by the authors, without undue reservation.

## References

[B1] Alexoudi A. Alexoudi I. Gatzonis S. (2018). Parkinson’s disease pathogenesis, evolution and alternative pathways: a review. Rev. Neurol. Paris. 174 (10), 699–704. 10.1016/J.NEUROL.2017.12.003 30131173

[B2] Alharbi B. A. (2020). Non-steroidal anti-inflammatory drugs and Parkinson’s disease: a systematic review and meta-analysis. Ann. Med. Health Sci. Res. 10 (5), 1023–1028.

[B3] Becker C. Jick S. S. Meier C. R. (2011). NSAID use and risk of Parkinson disease: a population-based case-control study. Eur. J. Neurol. 18 (11), 1336–1342. 10.1111/j.1468-1331.2011.03399.x 21457177

[B4] Bornebroek M. de Lau L. M. L. Haag M. D. M. Koudstaal P. J. Hofman A. Stricker B. H. C. (2007). Nonsteroidal anti-inflammatory drugs and the risk of Parkinson disease. Neuroepidemiology 28 (4), 193–196. 10.1159/000108110 17851257

[B31] Bower J. H. Maraganore D. M. Peterson B. J. (2006). Immunologic diseases, anti-inflammatory drugs, and Parkinson disease: a case-control study. Neurology. 67 (7109), 494–496. 16894114 10.1212/01.wnl.0000227906.99570.cc

[B5] Chen H. Jacobs E. Schwarzschild M. A. McCullough M. L. Calle E. E. Thun M. J. (2005). Nonsteroidal antiinflammatory drug use and the risk for Parkinson’s disease. Ann. Neurol. 58 (6), 963–967. 10.1002/ana.20682 16240369

[B6] Chen Z. F. Shi S. M. Hu R. X. Zhang M. Liang H. Zhou Z. Y. (2003). Nonsteroidal anti-inflammatory drugs and the risk of Parkinson disease. Arch. Neurol. 60 (8), 1059–1064. 10.1001/ARCHNEUR.60.8.1059 12925360

[B7] Driver J. A. Logroscino G. Lu L. Gaziano J. M. Kurth T. (2011). Use of non-steroidal anti-inflammatory drugs and risk of Parkinson’s disease: nested case-control study. BMJ 342, d198. 10.1136/bmj.d198 21252104 PMC3023971

[B30] Egger M. Smith G. D. Schneider M. Minder C. (1997). Bias in meta-analysis detected by a simple, graphical test. bmj. 315 (7109), 629–634. 9310563 10.1136/bmj.315.7109.629 PMC2127453

[B8] Etminan M. Carleton B. C. Samii A. (2008). Non-steroidal anti-inflammatory drug use and the risk of Parkinson disease: a retrospective cohort study. J. Clin. Neurosci. Off. J. Neurosurg. Soc. Australas. 15 (5), 576–577. 10.1016/j.jocn.2007.02.095 18343119

[B9] Etminan M. Suissa S. (2008). NSAID use and the risk of Parkinsons disease. Curr. Drug Saf. 1 (3), 223–225. 10.2174/157488606777934404 18690932

[B10] Gagne J. J. Power M. C. (2010). Anti-inflammatory drugs and risk of Parkinson disease: a meta-analysis. Neurology 74 (12), 995–1002. 10.1212/WNL.0b013e3181d5a4a3 20308684 PMC2848103

[B11] Hancock D. B. Martin E. R. Stajich J. M. Jewett R. Stacy M. A. Scott B. L. (2007). Smoking, caffeine, and nonsteroidal anti-inflammatory drugs in families with Parkinson disease. Arch. Neurol. 64 (4), 576–580. 10.1001/archneur.64.4.576 17420321

[B12] Herna M. A. Logroscino G. Garcı L. A. (2006). Inflammatory drugs and the incidence of Parkinson disease. Neurology 34, 1097–1099. 10.1212/01.wnl.0000204446.82823.28 16606925

[B13] Kabra A. Sharma R. Kabra R. Baghel U. S. (2018). Emerging and alternative therapies for Parkinson disease: an updated review. Curr. Pharm. Des. 24 (22), 2573–2582. 10.2174/1381612824666180820150150 30124146

[B14] Manthripragada A. D. Schernhammer E. S. Qiu J. Friis S. Wermuth L. Olsen J. H. (2011). Non-steroidal anti-inflammatory drug use and the risk of Parkinson’s disease. Neuroepidemiology 36 (3), 155–161. 10.1159/000325653 21508649 PMC3095838

[B15] Mirpour S. Turkbey E. B. Marashdeh W. El Khouli R. Subramaniam R. M. (2018). Impact of DAT-SPECT on management of patients suspected of parkinsonism. Clin. Nucl. Med. 43 (10), 710–714. 10.1097/RLU.0000000000002240 30153144

[B16] Pajares M. I Rojo A. Manda G. Boscá L. Cuadrado A. (2020). Inflammation in Parkinson’s disease: mechanisms and therapeutic implications. Cells 9 (7), 1687. 10.3390/CELLS9071687 32674367 PMC7408280

[B17] Parepally J. M. R. Mandula H. Smith Q. R. (2006). Brain uptake of nonsteroidal anti-inflammatory drugs: ibuprofen, flurbiprofen, and indomethacin. Pharm. Res. 23 (5), 873–881. 10.1007/s11095-006-9905-5 16715377

[B18] Poly T. N. Islam M. M. R. Yang H.-C. Li Y.-C. J. (2019). Non-steroidal anti-inflammatory drugs and risk of Parkinson’s disease in the elderly population: a meta-analysis. Eur. J. Clin. Pharmacol. 75 (1), 99–108. 10.1007/s00228-018-2561-y 30280208

[B19] Powers K. M. Kay D. M. Factor S. A. Zabetian C. P. Higgins D. S. Samii A. (2008). Combined effects of smoking, coffee, and NSAIDs on Parkinson’s disease risk. Mov. Disord. 23 (1), 88–95. 10.1002/mds.21782 17987647

[B20] Rathore C. Radhakrishnan A. Nayak S. D. Radhakrishnan K. (2009). Use of antihypertensives and the risk of Parkinson disease. Neurology 72 (6), 578–579. 10.1212/01.WNL.0000344171.22760.24 19204275

[B21] Samii A. Etminan M. Wiens M. O. Jafari S. (2009). NSAID use and the risk of Parkinson ’ s disease systematic review and meta-analysis of observational studies. Drugs Aging 26 (9), 769–779. 10.2165/11316780-000000000-00000 19728750

[B22] Sánchez-pernaute R. Ferree A. Cooper O. Yu M. Brownell A. Isacson O. (2004) Selective COX-2 inhibition prevents progressive dopamine neuron degeneration in a rat model of Parkinson ’ s disease, 11, 1–11. 10.1186/1742-2094-1-6 PMC483059 15285796

[B23] Starhof C. Hejl A.-M. Korbo L. Winge K. Friis S. (2020). Risk of multiple system atrophy and the use of anti-inflammatory drugs: a Danish register-based case-control study. Neuroepidemiology 54 (1), 58–63. 10.1159/000503003 31661696

[B24] Sung Y. F. Liu F. C. Lee J. T. Yang F. C. Chou Y. C. (2016). Reduced risk of Parkinson disease in patients with rheumatoid arthritis: a nationwide population-based study. Mayo Clin. Proc. 91 (10), 1346–1353. 10.1016/j.mayocp.2016.06.023 27712633

[B25] Terzi M. Altun G. Şen S. Kocaman A. Kaplan A. A. Yurt K. K. (2018). The use of non-steroidal anti-inflammatory drugs in neurological diseases. J. Chem. Neuroanat. 87, 12–24. 10.1016/j.jchemneu.2017.03.003 28341179

[B26] Ton T. G. Heckbert S. R. Longstreth W. T. Rossing M. A. Kukull W. A. Franklin G. M. (2006). Nonsteroidal anti-inflammatory drugs and risk of Parkinson’s disease. Mov. Disord. 21 (7), 964–969. 10.1002/mds.20856 16550541

[B27] Wahner A. D. Bronstein J. M. Bordelon Y. M. Ritz B. (2007). Nonsteroidal anti-inflammatory drugs may protect against Parkinson disease. Neurology 69 (19), 1836–1842. 10.1212/01.wnl.0000279519.99344.ad 17984451

[B28] Wang T. Pei Z. Zhang W. Liu B. Langenbach R. Lee C. (2005). “MPP + -induced COX-2 activation and subsequent dopaminergic neurodegeneration,” vol. 18, pp. 1134–1136. 10.1096/fj.04-2457fje 15845609

[B29] Zafar S. Yaddanapudi S. S. (2022). Parkinson disease. StatPearls 8, 1–13. 29261972

